# Semaphorin 3F expression is reduced in pregnancy complicated by preeclampsia. An observational clinical study

**DOI:** 10.1371/journal.pone.0174400

**Published:** 2017-03-28

**Authors:** Giovanni Stallone, Maria Matteo, Giuseppe Stefano Netti, Barbara Infante, Adelaide Di Lorenzo, Clelia Prattichizzo, Stefania Carlucci, Federica Trezza, Loreto Gesualdo, Pantaleo Greco, Giuseppe Grandaliano

**Affiliations:** 1 Nephrology Dialysis and Transplantation Unit, Dept. of Medical and Surgical Sciences, University of Foggia, Viale Luigi Pinto, 1, Foggia, Italy; 2 Gynaecologic and Obstetric Unit, Dept. of Medical and Surgical Sciences, University of Foggia, Viale Luigi Pinto, 1, Foggia, Italy; 3 Clinical Pathology Unit, Dept. of Medical and Surgical Sciences, University of Foggia, Viale Luigi Pinto, 1, Foggia, Italy; 4 Nephrology Dialysis and Transplantation Unit, Dept. of Emergency and Organ Transplantation, University of Bari “A. Moro”, Piazza G. Cesare 11, Bari, Italy; VU medisch centrum, NETHERLANDS

## Abstract

**Background and objective:**

Preeclampsia is a systemic disorder, affecting 2–10% of pregnancies, characterized by a deregulated pro- and anti-angiogenic balance. Semaphorin 3F is an angiogenesis inhibitor. We aimed to investigate whether semaphorin 3F expression is modulated in preeclampsia.

**Design, setting, participants, and measurements:**

We performed two observational single center cohort studies between March 2013 and August 2014. In the first we enrolled 110 consecutive women, undergoing an elective cesarean section; in the second we included 150 consecutive women undergoing amniocentesis for routine clinical indications at 16–18 week of gestation. Semaphorin 3F concentration was evaluated in maternal peripheral blood, venous umbilical blood and amniotic fluid, along with its placenta protein expression at the time of delivery in the first study group and in the amniotic fluid at 16–18 weeks of gestation in the second study group.

**Results:**

In the first study 19 patients presented at delivery with preeclampsia. Semaphorin 3F placenta tissue expression was significantly reduced in preeclampsia. In addition, semaphorin 3F level at delivery was significantly lower in serum, amniotic fluid and venous umbilical blood of preeclamptic patients compared with normal pregnant women. In the prospective cohort study 14 women developed preeclampsia. In this setting, semaphorin 3F amniotic level at 16–18 weeks of gestation was reduced in women who subsequently developed preeclampsia compared to women with a normal pregnancy. ROC curve analysis showed that semaphorin 3F amniotic levels could identify women at higher risk of preeclampsia.

**Conclusions:**

Semaphorin 3F might represent a predictive biomarker of preeclampsia.

## Introduction

Preeclampsia is a systemic disorder, characterized by the new onset of hypertension and proteinuria after 20 weeks of gestation. This condition, unique to humans, complicates 2–10% of pregnancies worldwide (1). Preeclampsia recognizes two key pathogenic events: impaired placenta implantation and subsequent systemic endothelial dysfunction [1]. Indeed, placenta delivery resolves the acute clinical signs of preeclampsia, suggesting that placenta vascular bed plays a central role in the pathogenesis of this condition. A fine balance between pro- and anti-angiogenic factors is a key feature of normal pregnancy [2]. Preeclampsia, on the other hand, is characterized by an ineffective remodeling of maternal vessels perfusing the placenta. Although reduced angiogenesis is often considered a hallmark of this pathological condition, different observations suggest an increased angiogenesis in preeclamptic placenta [3].

Semaphorins are a large and extremely heterogeneous family of soluble and membrane-associated proteins ordered into eight classes. Semaphorins are widely expressed in several tissues, although their expression patterns are best characterized in the nervous and cardiovascular system. Semaphorins expression profiles have been recently described in immune system, kidney and in different types of malignancies [4].

Semaphorin 3F is a tumor suppressor. The gene encoding for this protein was identified in a region of human chromosome 3p21.3, commonly deleted in small cell lung carcinoma [5]. Semaphorin 3F is a powerful anti-angiogenic factor, inhibiting vascular endothelial growth factor (VEGF)-induced proliferation of human umbilical vein endothelial cells [6,7]. Plexines are the main semaphorins’ receptors, but semaphorin 3 interacts also with another class of membrane-associated proteins, the Neuropilines (NRP). NRP1 and NRP2 are single pass trans-membrane glycoproteins, first identified in neurons [8]. NRP1 binds both semaphorin 3A and 3F, but with a higher affinity for the latter, while NRP2 binds semaphorin 3F only [9]. NRPs are expressed by arterial, venous and lymphatic endothelium [10].

On this basis, we investigated whether semaphorin 3F might be involved in the development of late onset preeclampsia. In addition, we evaluated whether its concentrations in the amniotic fluid at the 16–18 weeks of pregnancy may represent a predictive biomarker of the disease.

## Subjects and methods

### Study population

The study was approved by the University of Foggia Ethical Committee, (IRB protocol number 53/EC/2013). Each woman enrolled in both studies signed a written informed consent approved by the Ethics Committee.

We performed two single center observational studies, in the period from March 2013 to August 2014. In the first study we included 110 consecutive pregnant women undergoing an elective cesarean section, meeting the following inclusion and exclusion criteria. Inclusion criteria were: elective caesarean section at our Obstetric and Gynecologic Unit; no previous pregnancy; age >18 years; written informed consent form. Exclusion criteria were: history of venous thromboembolism; twin pregnancy; early onset preeclampsia; presence of an autoimmune disease (systemic lupus erythematous, anti-phospholipid syndrome); any other systemic disease, including chronic hypertension, diabetes mellitus, cardiomyopathy, liver diseases, chorioamnionitis, infectious diseases. Out of 110 patients enrolled, 19 presented with preeclampsia. Preeclampsia was diagnosed according to the International Society for the Study of Hypertension in Pregnancy (ISSHP) criteria [11]. Samples of maternal peripheral blood, fetal cord blood and amniotic fluid were collected at the time of delivery. Moreover, we collected a placental cotyledon, close to umbilical cord. We included in the study a control group of 40 non-pregnant healthy women.

In the same period of time, we enrolled a second group of 150 consecutive pregnant women undergoing amniocentesis, for age (>35 years old) or for patients request, at 16–18 week of gestation and meeting the inclusion/exclusion criteria indicated for the first group of patients. In this second cohort we measured semaphorin 3F levels in the amniotic fluid at the time of amniocentesis. To this purpose we used the supernatant of amniotic fluid remained after clinical testing, including karyotype analysis and α-fetoprotein measurement. Fourteen out of 150 women of this group developed late preeclampsia according to the ISSHP criteria [11].

### Samples collection

Three placenta tissue samples/patient were collected using a sterile scalpel within 10 minutes of placenta delivery. The samples were removed from a medio-basal location as defined by Wyatt el al [12]. Each tissue sample was washed three times with ice-cold PBS and then snap-frozen in Optimal Cutting Temperature medium (OCT, Sakura Finetek Europe BV, Leiden, The Netherland) and stored at -80°C until analyzed. PBS after washing was collected and stored at -80°C to detect possible release of semaphorin 3F from syncytium trophoblast. Maternal serum, cord blood and amniotic fluid samples were collected from all patients and stored at -80°C until used.

### Confocal microscopy

Confocal microscopy was performed on 5-μm thick cryostat tissue sections from both normal and preeclamptic placentas using a confocal laser-scanning microscope (TCS SP5, Leica Microsystems AG, Wetzlar, Germany). All the reagents were prepared in 0.05% TritonX100-containing PBS to permeabilize cell membranes. Staining with primary goat polyclonal anti-human semaphorin 3F IgG antibody (clone G-14, Santa Cruz Biotechnologies, Santa Cruz, CA), CD31 monoclonal antibody (clone JC70, Ventana Medical Systems, Tucson, AZ), NRP2 monoclonal antibody (clone C-9, Santa Cruz Biotechnologies, Santa Cruz, CA), HIF-1 alpha monoclonal antibody (H1alpha 67, Santa Cruz Biotechnologies) and secondary Alexa Fluor 488-labeled rabbit anti-goat IgG, Alexa Fluor 546-labeled goat anti-mouse IgG, Alexa Fluor 488-labeled goat anti-mouse IgG respectively (Molecular Probes, Eugene, OR) were performed following the manufacturers’ instructions. Nuclei were counterstained with Topro-3 (Molecular Probes). Specific fluorescence quantification was performed as previously described [13].

### Protein extraction and western blot assay

Western blot studies were performed using all the placenta samples from the preeclampsia group and 19 randomly selected samples from the control group. To this purpose, tissue samples (200 mg) were homogenized in protein extraction buffer (Tris-HCl, NP-40, NaCl, EDTA, NaN3, and PMSF at pH 7.5) with freshly added DTT, leupeptin and aprotinin (protease inhibitors). The lysate was centrifuged at 14,000xg for 15 min at 4°C. The supernatant was collected and protein concentration was measured by the Bradford method (Bio-Rad Laboratories, Hercules, CA). Proteins were separated by SDS-PAGE (10% polyacrylamide) and electro-transferred onto PVDF membrane (Millipore, Bedford, MA). The filters were blocked with 5% non-fat milk powder in PBS containing 0.1% TWEEN-20 (TBS), incubated with anti-semaphorin 3F (1:250) and NRP2 monoclonal antibody (1:250), washed again in T-PBS, incubated with horseradish peroxidase-conjugated secondary antibodies (rabbit anti-goat and goat anti-mouse IgG 1:3000 respectively, Bio-Rad Laboratories) and the signal was detected by the ECL-enhanced chemiluminescence system (Amersham, Piscataway, NJ). The image was acquired using a scanner UVP Chemidoc-IT (UVP, LLC, Upland, CA) and quantified by NIH ImageJ software. Membranes were stripped; immunoblotted with anti-β-actin antibody (1:10000 in TBS) and β-actin bands intensity was used to control for equal loading.

### ELISA

Semaphorin 3F serum and amniotic fluid levels were measured by ELISA using a commercially available kit, following manufacturer’s instructions (USCN Life Science Inc, Houston, TX), with a mean minimum detectable concentration of 17.55 pg/ml and a maximum detectable concentration of 3000 pg/ml. Semaphorin 3F levels in PBS collected after washing of placental tissue samples were assayed by the same kit. All serum and amniotic fluid samples were diluted 1:1000 and 1:100, respectively, while washing PBS was assayed undiluted. All the samples were assayed in duplicate.

### Statistical analysis

Statistical analyses were performed using the SPSS software (SPSS 17.0 Inc., Evanston, IL). Continuous variables were compared by paired or unpaired Student t-test or Mann-Whitney *u* test, as appropriate. Correlation between two variables was determined by linear regression analysis and Spearman's correlation test as appropriate. A Receiver operator characteristic curves (ROC) analysis was performed and an operational cut-off level was defined in order to identify pregnant women at risk to develop preeclampsia. A two-sided p<0.05 was considered statistically significant. Results were expressed in the text as mean ± standard deviation (SD) unless otherwise stated.

## Results

The main clinical features of mothers and newborns at delivery from the first group are shown in Table 1. Newborns from preeclamptic women at delivery had lower gestational age and birth weight (Table 1). Also placental weight was lower in women with preeclampsia compared with normal women (Table 1).

**Table 1:** Main clinical features of mothers and newborns at delivery. The values are expressed as median (25^th^-75^th^ percentile).

	Normal pregnants (n = 91)	Preeclamptic patients (n = 19)	*p*
**Age (years)**	33 (30–37)	37 (31.5–40.5)	ns
**Gestational Age (weeks)**	38.4 (38.2–38.6)	35.6 (32.2–38.5)	<0.01
**Newborn weight (g)**	3100 (2880–3400)	1850 (1490–2810)	<0.001
**Placental weight (g)**	620 (575–670)	435 (340–506)	<0.001
**Mean Arterial Blood Pressure (mmHg)**	85 (75–90)	95 (80–105)	ns
**Body Mass Index (m**^**2**^**/kg)**	23.5 (21.5–26.0)	24.5 (22.0–27.5)	ns

### Semaphorin 3F expression is reduced in placental tissues of preeclamptic patients at delivery

**Fig 1:**
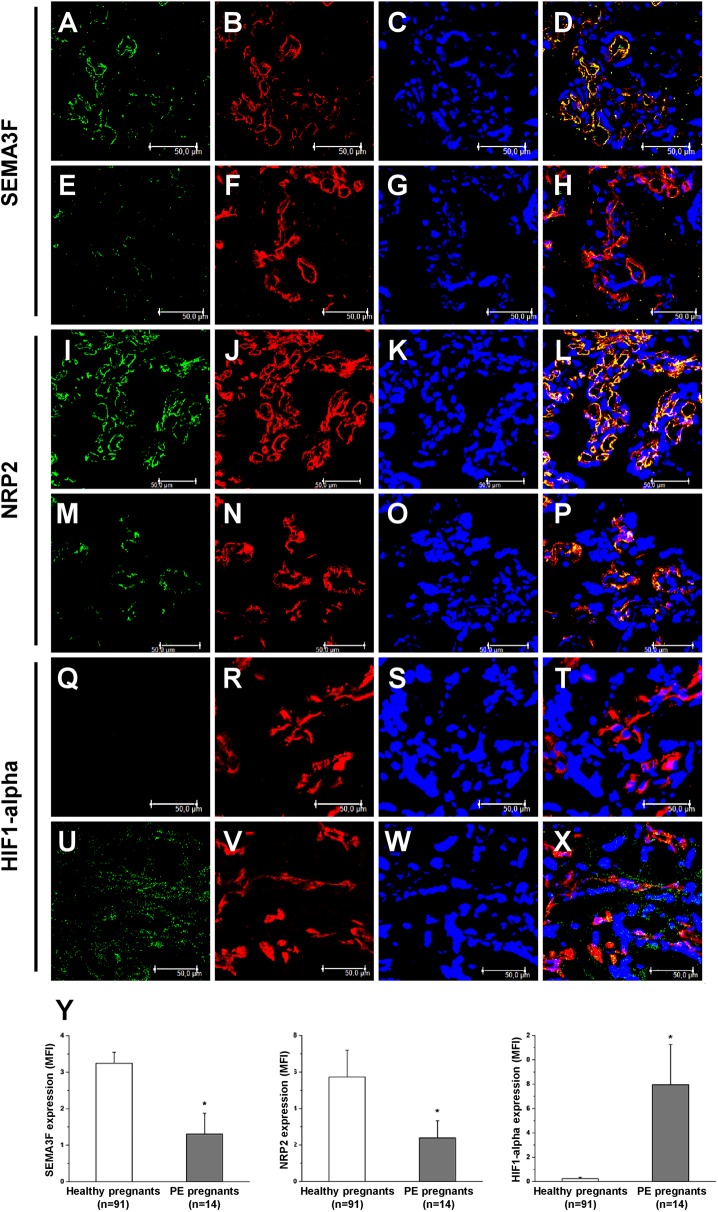
Semaphorin 3F, NRP2 and HIF1-alpha expression in placental tissues. Double-label immunofluorescence showing expression of semaphorin 3F (green) and CD31 (red) in the placenta of both normal (A-D) and preeclamptic (E-H) patients. To-pro-3 counter stains nuclei (blue). Merged images (yellow) demonstrate that semaphorin 3F was more expressed by CD31-positive endothelial cells in normal patients (A-D) than in preeclamptic patients (E-H). Double-label immunofluorescence showing expression of NRP2 (green) and CD31 (red) in the placenta of both normal (I-L) and preeclamptic (M-P) patients. To-pro-3 counter stains nuclei (blue). Merged images (yellow) demonstrate that NRP2 was highly expressed at endothelial level (I-L), while its expression was strongly reduced in preeclamptic placentae (M-P). Double-label immunofluorescence showing expression of HIF1 alpha (green) and CD31 (red) in the placental tissue of both normal (Q-T) and preeclamptic (U-X) patients. To-pro-3 counter stains nuclei (blue). Merged images (yellow) demonstrate that HIF1 alpha was absent in normal placentae (Q-T), while its expression was significantly increased in preeclamptic placentae (U-X). Specific semaphorin 3F immunofluorescence quantification (Y, left histogram) showed statistically significant difference in mean fluorescence intensity (MFI) between normal and preeclamptic patients. Quantification of specific NRP2 fluorescence (Y, center histogram) demonstrated that MFI of NRP2 in normal placentae was significantly higher than in preeclamptic placentae. Quantification of specific fluorescence (Y, right histogram) confirmed the absence of HIF1 alpha signal in normal placentae and its increased expression in preeclamptic placentae. Results are representative of 10 patients. (*) p<0.001.

Semaphorin 3F was constitutively expressed by the endothelial cells of chorionic villi in normal placenta (Fig 1A–1D), while its placenta expression was markedly reduced in patients with preeclampsia (Fig 1E–1H). Specific semaphorin 3F immunofluorescence quantification (Fig 1Y, left histogram) confirmed a statistically significant difference in MFI between normal and preeclamptic placenta (3.2±0.3 vs. 1.3±0.6 Arbitrary Units, p<0.001).

To rule out a possible release of semaphorin 3F from syncytium trophoblast during washing of placental cotyledons at time of collection, the presence of semaphorin 3F in the washing solution was assayed, by ELISA, and resulted absent.

We, then, investigated, by confocal microscopy, the placenta expression of specific semaphorin 3F receptor NRP2. In normal placenta NRP2 was highly expressed at the endothelial level (Fig 1I–1L), while its expression was strongly reduced in preeclamptic placenta (Fig 1M–1P). Specific NRP2 immunofluorescence quantification (Fig 1Y, center histogram) confirmed that NRP2 expression in normal placenta was significantly higher than in preeclamptic placenta (5.7±1.5 vs. 2.4±0.9 AU, p<0.001).

The down-regulation of anti-angiogenic signals semaphorin 3F and NRP2 in preeclamptic placenta was strictly associated with tissue ischemia, as suggested by the increased expression in preeclamptic placental tissues of Hypoxia-inducible factor 1-alpha (HIF1-alpha) (Fig 1U–1X), which was almost absent in normal placenta (Fig 1Q–1T). Specific HIF1-alpha immunofluorescence quantification (Fig 1Y, right histogram) confirmed a low level of HIF1-alpha signal in normal placenta, while its expression was strongly elevated in preeclamptic placenta (0.2±0.1 vs. 7.9±3.3 AU, p<0.001). Noteworthy, linear regression analysis of specific immunofluorescence quantification in preeclamptic placenta showed that HIF1-alpha signal was inversely and significantly correlated to semaphorin 3F expression (R^2^ = 0.675; p = 0.01).

**Fig 2:**
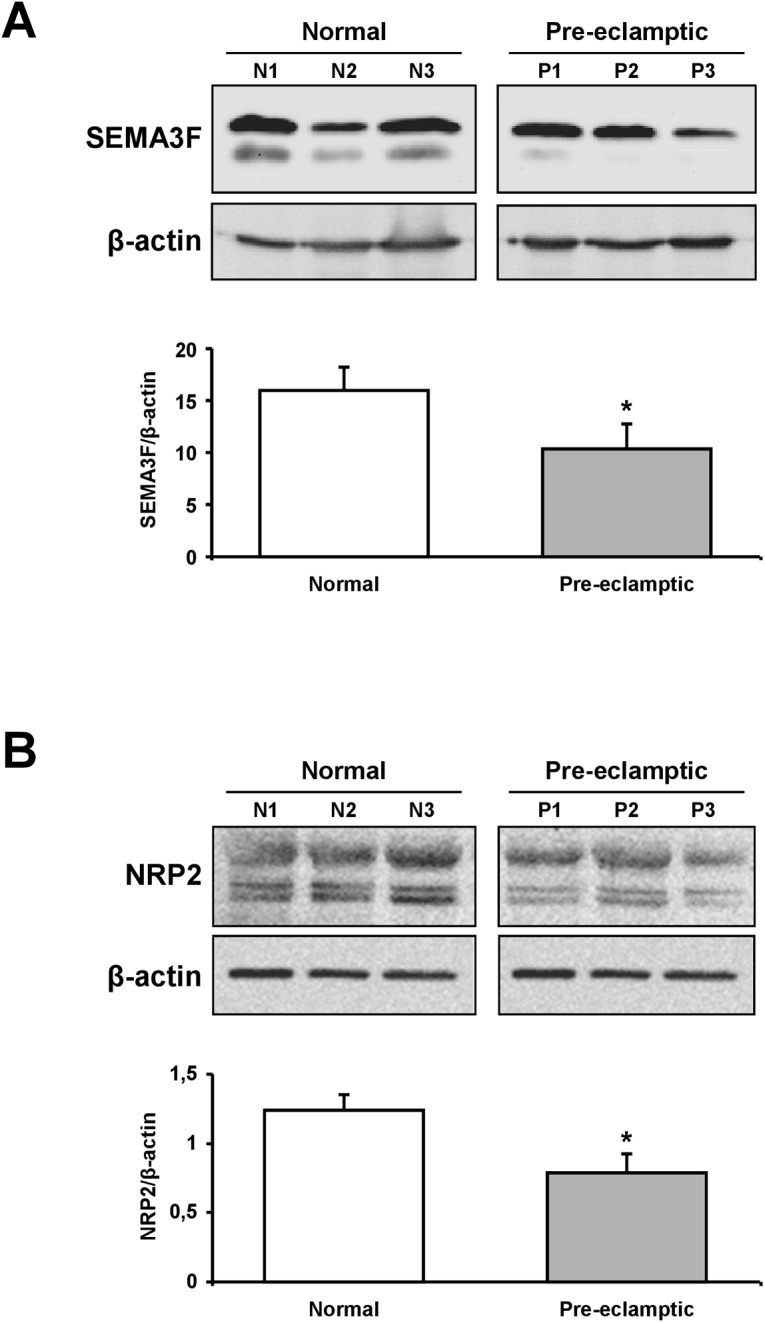
Semaphorin 3F and NRP2 protein expression in placental tissues. Semaphorin 3F (A) and NRP2 (B) protein expression was analyzed by immunoblotting in placental tissues from both normal and preeclamptic subjects. Semaphorin 3F and NRP2 protein expression was significantly higher in normal placentae as compared to preeclamptic placentae. Quantification of semaphorin 3F and NRP2 protein expression is normalized to β-actin bands intensity. Data are represented as mean semaphorin 3F/β-actin and NRP2/β-actin ratio ± SD and are representative of the whole preeclampsia group and of 19 randomly selected women from the control group. (*) p = 0.04 for Semaphorin 3F and p = 0.03 for NRP2, respectively.

Protein expression of semaphorin 3F and NRP2 was also evaluated by immunoblotting. This semi-quantitative approach confirmed that both semaphorin 3F and NRP2 were significantly higher in normal as compared to preeclamptic placenta (Fig 2A and 2B)

### Semaphorin 3F levels are reduced in serum, amniotic fluid and cord blood of patients with preeclampsia at delivery

**Fig 3:**
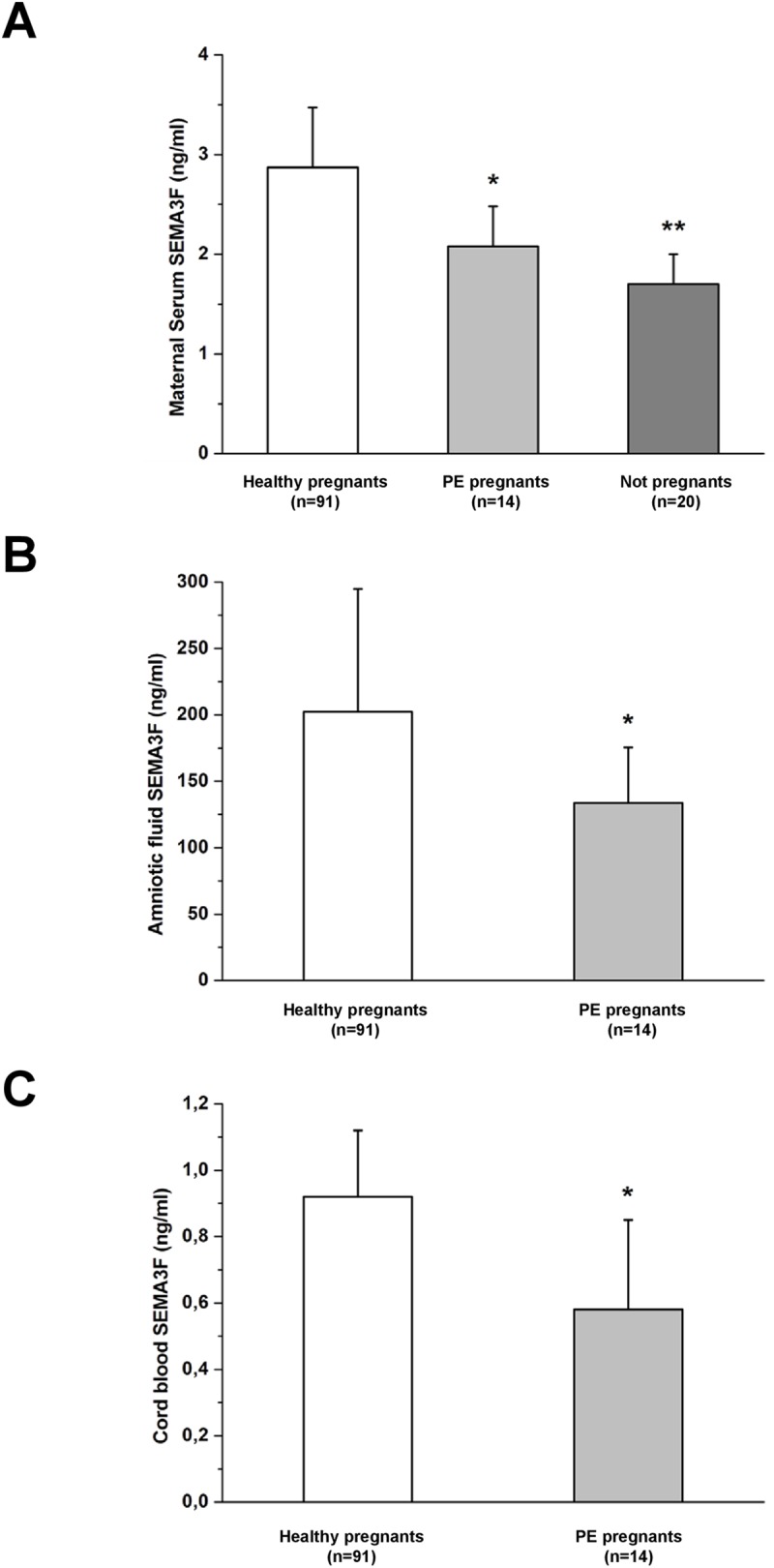
Semaphorin 3F levels in maternal serum, cord blood and amniotic fluid at delivery. Semaphorin 3F levels were significantly higher in normal pregnant (n = 91) in comparison with preeclampsia patients (n = 19) in maternal serum (A), cord blood (B) and amniotic fluid (C) at delivery (2.01±0.33 vs. 2.92±0.55 ng/mL for serum samples, p<0.001; 131.70±36.20 vs. 198.18±83.51 ng/mL for amniotic fluid, p = 0.001; 0.58±0.27 vs. 0.92±0.20 ng/mL for cord blood, respectively, p = 0.038). Serum semaphorin 3F levels were also assessed in a cohort of non-pregnant health female controls (n = 40), which resulted comparable to preeclampsia patients (1.70±0.30 vs. 2.01±0.33 ng/mL, p = 0.06). (*) p<0.001 vs. normal women; (**) p<0.001 vs. normal women and p = 0.06 vs preeclampsia women; (^#^) p = 0.02 vs. normal women.

To confirm our data on lower semaphorin 3F expression in preeclamptic women, we assessed its levels in maternal serum, amniotic fluid and cord blood at delivery and we observed that the levels of this anti-angiogenic factor were significantly lower in samples from preeclamptic patients as compared with those from normal pregnant women (2.01±0.33 vs. 2.92±0.55 ng/mL in maternal serum, p<0.001; 131.70±36.20 vs. 198.18±83.51 ng/mL in amniotic fluid, p = 0.001; 0.58±0.27 vs. 0.92±0.20 ng/mL in cord blood, respectively; p = 0.03) (Fig 3A–3C)

Noteworthy, serum semaphorin 3F levels in patients with preeclampsia were comparable to those of non-pregnant health female controls (n = 40) (2.01±0.33 vs. 1.70±0.30 ng/mL, p = 0.07) (Fig 3A). Interestingly, semaphorin 3F concentrations both in maternal serum and in amniotic fluid were significantly associated with placental weight and newborn weight. In detail, linear regression analysis demonstrated a strong association between semaphorin 3F levels in maternal serum and both placental weight (R^2^ = 0.8299; p<0.001) and newborn weight (R^2^ = 0.5563; p<0.001) at birth. The same relationship was observed by linear regression analysis between semaphorin 3F levels in amniotic fluid and both placental weight (R^2^ = 0.4889; p<0.001) and newborn weight (R^2^ = 0.2872; p<0.001) at birth.

### Semaphorin 3F levels are reduced in amniotic fluid at 16–18 weeks of gestation in pregnant women developing preeclampsia

We investigated the levels of semaphorin 3F in the amniotic fluid at 16–18 weeks of gestation. Semaphorin 3F levels were measured in the amniotic fluid from 150 healthy pregnant women undergoing amniocentesis for routine clinical indications. All the pregnant women were, then, followed until the delivery and the main clinical outcome were recorded. The main clinical characteristics of the entire study group are summarized in Table 2.

**Table 2:** Main clinical features of pregnant women at amniocentesis. The values are expressed as median (25^th^-75^th^ percentile).

	Pregnant women undergoing amniocentesis (n = 150)	Pregnant women not developing PE (n = 136)	Pregnant women developing PE (n = 14)	*p*
**Age (years)**	36 (35–39)	36 (35–38)	37 (35–41)	ns
**Gestational Age (weeks)**	17 (16–18)	17 (16–18)	17 (16–18.5)	ns
**Mean Arterial Blood Pressure (mmHg)**	85 (75–95)	85 (75–90)	90 (85–95)	ns
**Body Mass Index (m**^**2**^**/kg)**	22.5 (21.5–24.0)	22.5 (21.5–24.0)	23.0 (22.0–24.5)	ns

**Fig 4:**
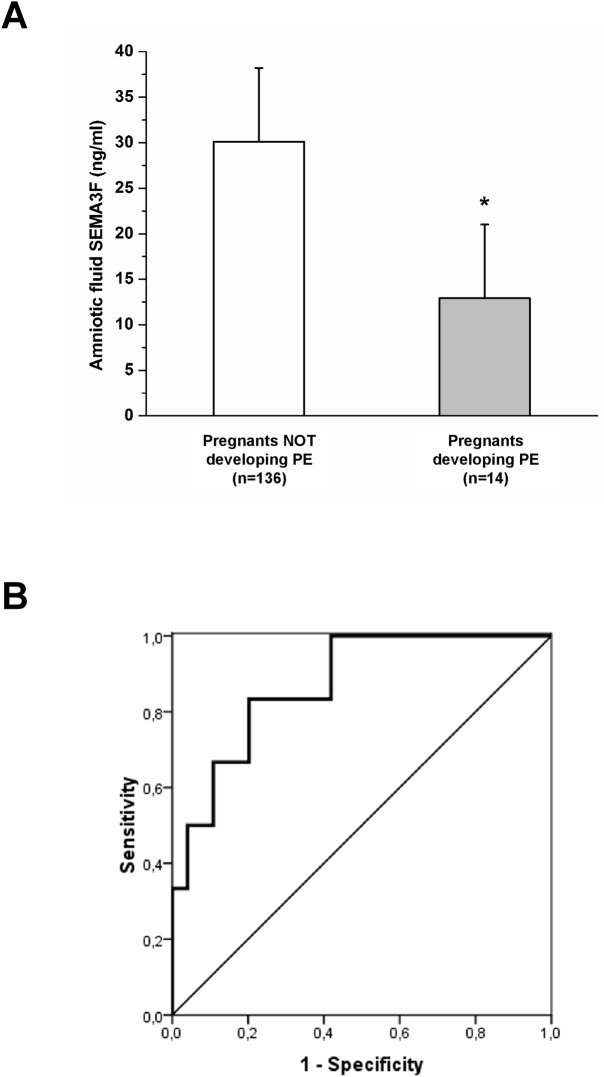
Semaphorin 3F levels in amniotic fluid at amniocentesis. (A) Quantification of semaphorin 3F levels at amniocentesis showed significantly lower levels in the amniotic fluid in pregnant women subsequently developing preeclampsia as compared with women carrying out a normal pregnancy (9.08±7.7 vs. 30.85±15.81 ng/mL, p<0.001). (B) The ROC curve analysis confirmed the reliability of semaphorin 3F amniotic fluid levels to identify women at higher risk to develop preeclampsia (AUC 0.941, p<0.001) with a cut-off value of 18.35 ng/mL (B). (*) p<0.001.

Among the entire study group, 14 patients, who developed late preeclampsia, showed significantly lower semaphorin 3F levels in the amniotic fluid at amniocentesis as compared with women who carried out a normal pregnancy (9.08±7.7 vs. 30.85±15.81 ng/mL, p<0.001) (Fig 4A)

A ROC curve analysis was performed to further validate the role of semaphorin 3F amniotic levels at amniocentesis in predicting the risk of preeclampsia onset and to define an operational cut-off value. The analysis demonstrated that semaphorin 3F amniotic levels were able to reliably identify pregnant women at higher risk to develop preeclampsia (AUC 0.941, p<0.001), and to define a cut-off value of 18.35 ng/mL with a 80.1% specificity and a 90.9% sensitivity, a positive predictive value of 32.5% and a negative predictive value of 99.1% (Fig 4B)

## Discussion

In the present study we demonstrate for the first time that semaphorin 3F placenta tissue expression is significantly reduced in women with preeclampsia, along with its levels in maternal serum, amniotic fluid and venous umbilical blood at the time of delivery. In addition, a decreased semaphorin 3F concentration in the amniotic fluid was already present at 16–18 weeks of gestation, potentially suggesting this anti-angiogenic factor as a predictive biomarker of preeclampsia.

Angiogenesis is a multifaceted biological process controlled by several factors, directly or indirectly, promoting or inhibiting endothelial cells growth and migration. Maynard et al. [14] were the first to observe that preeclampsia is characterized by the presence of anti-angiogenic signals involving the up-regulation of placenta-derived soluble fms-like tyrosine kinase-1 (sFlt-1) in maternal plasma. This soluble receptor antagonizes the angiogenic effects of VEGF and placental growth factor. Subsequently, others authors confirmed the presence of an anti-angiogenic state in preeclampsia as well as in other pregnancy-related disorders [15,16]. Indeed, there is a growing body of evidence that anti-angiogenic mediators in maternal serum may serve as potential biomarkers for adverse pregnancy outcomes [17,18].

On the other hand, the extra-villous trophoblast displays a phenotype very similar to cancer cells, with the ability to proliferate, migrate, induce angiogenesis and immune tolerance, using similar molecular mechanisms [19,20]. The *conditio sine qua non* of both a successful pregnancy and malignancy development and progression is the establishment of an adequate oxygen and nutrient supply, and invasion through normal tissues is essential for this process. Cell invasion requires changes in the expression of adhesion molecules, secretion of proteases and availability of cytokine and growth factors [20]. NRPs expression is modulated in this process [21]. Coma et al [22] report that cancer cells repress NRP2 transcription under hypoxic conditions. NRP2 down-regulation in tumor cells significantly influenced the biological activities of its two ligands, semaphorin 3F and VEGF while inducing an increase of VEGF protein levels in conditioned media, resulting in increased paracrine activation of endothelial cells. These results, suggesting a novel mechanism linking hypoxia to neoplastic angiogenesis, might also explain the down-regulation of NRP-2 observed in our setting [22].

The reduced invasiveness of extra-villous trophoblast in preeclampsia is considered to be due to an increased placenta expression and release of sFlt-1 [23–25]. sFLT-1, a soluble VEGF receptor, binding to its ligand specifically inhibits VEGF activity and its excessive production leads to a reduced angiogenic potential. In this setting, it is surprising to find a clear reduction in the expression of semaphorin 3F, a well-known anti-angiogenic mediator. Interestingly, semaphorin 3F has been shown to directly disrupt VEGF signaling [3]. Thus, it is conceivable that, in the face of a reduced VEGF signaling, the physiological response might be represented by the reduction in one of the natural anti-VEGF factors. We hypothesized that placenta ischemia, featuring preeclampsia, might induce the down-regulation of the anti-angiogenic system represented by semaphorin 3F and its receptor, NRP-2, in the attempt to improve placenta angiogenesis and blood supply. This hypothesis is supported by our observation of a clear association between the reduction in semaphorin 3F gene expression and the significant increase in HIF-1-alpha protein expression within the preeclamptic placenta. HIF-1alpha, in fact, is a critical mediator of the hypoxic response [26–28].

The reduced expression of a powerful anti-angiogenic factor should inevitably lead to an increased angiogenesis. Interestingly, there are several observations suggesting that preeclampsia is characterized, indeed, by an excessive angiogenesis. Escudero et al. observed a significantly increase in the number of CD31-positive cells within the diffusion villi and a higher CD31 and CD34 protein level in placentas from preeclamptic women in comparison with those from normal pregnancies [3]. Resta et al reported a dramatic increase in the ramification of the capillary loops, characterized by irregular profile and narrow lumina, within preeclamptic placentas [29]. Since the neoangiogenic response to ischemia in this setting appears extremely deregulated, this process, instead of preventing placenta ischemia may even further reduce the perfusion of the feto-placental unit. Indeed, our data on the correlation between semaphorin levels and placenta as well as newborn weight confirm this hypothesis and are supported by recently findings in which the down-regulation of well-known pro-angiogenic factor is correlated with fetal growth restriction [30].

To date no specific mechanisms has been suggested to explain the angiogenic response in preeclampsia. Interestingly, Escudero et al observed that placenta angiogenesis in this setting was not associated with an increased VEGF receptor expression, but with a significant activation of its downstream signaling [3]. This observation along with our findings might suggest a role for semaphorin 3F in this context since this soluble mediator is known to interfere with VEGF signaling. Finally, if we accept the hypothesis linking semaphorin 3F expression with placenta ischemia, our observation of a significant reduction of semaphorin 3F levels in the amniotic fluid at 16–18 weeks of gestation might directly support the hypothesis that placenta hypo-perfusion in preeclampsia develops in the first weeks of pregnancy. In addition, this observation might also suggest a role for semaphorin 3F as an early predictive biomarker of preeclampsia.

A potential limitation of our study might be represented by the relatively small sample size of the perspective cohort, although this part of the study should be regarded as a further confirmation of the semaphorin 3F role in the development of preeclampsia. Conversely, the evaluation of semaphorin 3F expression on placenta tissue, and the correlation between its serum and amniotic fluid levels with the main clinical feature of pregnancy, as well as the results from our prospective cohort, certainly represent key strengths of our study.

In conclusion, the present study demonstrates for the first time that an anti-angiogenic system, including semaphorin 3F and its receptor NRP2, is significantly reduced in preeclamptic placenta. In addition, our findings support the hypothesis that amniotic fluid levels of semaphorin 3F might represent a predictive biomarker of preeclampsia.

## Supporting information

S1 Fig Western blot for SEMA3F and NRP2 on normal vs. PE placental tissues. Original uncropped and unadjusted blots of SEMA3F and NRP2, as shown in Fig 2A and 2B.
(PPTX)

## References

[1] Sibai B, Dekker G, Kupferminc M. Pre-eclampsia. Lancet. 2005; 365: 785–799. 10.1016/S0140-6736(05)17987-2 15733721

[2] Grill S, Rusterholz C, Zanetti-Dällenbach R, Tercanli S, Holzgreve W, Hahn S, et al Potential markers of preeclampsia-a review. Reprod Biol Endocrinol. 2009; 7: 70–78. 10.1186/1477-7827-7-70 19602262 PMC2717076

[3] Escudero C, Celis C, Saez T, San Martin S, Valenzuela FJ, Aguayo C, et al Increased placental angiogenesis in late and early onset pre-eclampsia is associated with differential activation of vascular endothelial growth factor receptor 2. Placenta. 2014; 35: 207–215. 10.1016/j.placenta.2014.01.007 24508097

[4] Yazdani U, Terman JR. The semaphorins. Genome Biology. 2006; 7: 211–221. 10.1186/gb-2006-7-3-211 16584533 PMC1557745

[5] Roche J, Boldog F, Robinson M, Robinson L, Varella-Garcia M, Swanton M, et al Distinct 3p21.3 deletions in lung cancer and identification of a new human semaphorin. Oncogene. 1996; 12: 1289–1297. 8649831

[6] Kessler O, Shraga-Heled N, Lange T, Gutmann-Raviv N, Sabo E, Baruch L, et al Semaphorin-3F Is an Inhibitor of Tumor Angiogenesis. Cancer Res. 2004; 64: 1008–1015. 14871832 10.1158/0008-5472.can-03-3090

[7] Bielenberg DR, Hida Y, Shimizu A, Kaipainen A, Kreuter M, Kim CC, et al Semaphorin 3F, a chemorepulsant for endothelial cells, induces a poorly vascularized, encapsulated, nonmetastatic tumor phenotype. J Clin Invest. 2004; 114: 1260–1271. 10.1172/JCI21378 15520858 PMC524226

[8] Takagi S, Tsuji T, Amagai T, Akamatsu T, Fujisawa H. Specific cell surface labels in the visual centers of Xenopus laevis tadpole identified using monoclonal antibodies. Dev Biol. 1987; 122: 90–100. 3297854 10.1016/0012-1606(87)90335-6

[9] Chen H, Chedotal A, He Z, Goodman CS, Tessier-Lavigne M. Neuropilin-2, a novel member of the neuropilin family, is a high affinity receptor for the semaphorins Sema E and Sema IV but not Sema III. Neuron. 1997; 19: 547–559. 9331348 10.1016/s0896-6273(00)80371-2

[10] Geretti E, Klagsbrun M. Neuropilins Novel Targets for Anti-Angiogenesis Therapies. Cell Adhes Migr. 2007; 2: 56–61. 10.4161/cam.1.2.4490 PMC2633972 19329879

[11] Brown MA, Lindheimer MD, de Swiet M, van Assche A, Moutquin JM. The classification and diagnosis of the hypertensive disorders of pregnancy: statement from the International Society for the Study of Hypertension in Pregnancy (ISSHP). Hypertens Pregnancy. 2001; 20: IX–XIV. 10.1081/PRG-100104165 12044323

[12] Wyatt SM, Kraus FT, Roh CR, Elchalal U, Nelson DM, Sadovsky Y. The correlation between sampling site and gene expression in the term human placenta. Placenta. 2005; 26: 372–379. 10.1016/j.placenta.2004.07.003 15850641

[13] Stallone G, Cormio L, Netti GS, Infante B, Selvaggio O, DiFino GD, et al Pentraxin 3: a novel biomarker for predicting progression from prostatic inflammation to prostate cancer. Cancer Res. 2014; 74: 4230–4238. 10.1158/0008-5472.CAN-14-0369 24950910

[14] Maynard SE, Min JY, Merchan J, Lim KH, Li J, Mondal S, et al Excess placental soluble fms-like tyrosine kinase 1 (sFlt1) may contribute to endothelial dysfunction, hypertension, and proteinuria in preeclampsia. J Clin Invest. 2003; 111: 649–658. 10.1172/JCI17189 12618519 PMC151901

[15] Romero R, Nien JK, Espinoza J, Todem D, Fu W, Chung H, et al A longitudinal study of angiogenic (placental growth factor) and anti-angiogenic (soluble endoglin and soluble vascular endothelial growth factor receptor 1) factors in normal pregnancy and patients destined to develop preeclampsia and deliver a small for gestational age neonate. J Matern Fetal Neonatal Med. 2008; 21: 9–23. 10.1080/14767050701830480 18175241 PMC2587364

[16] Chaiworapongsa T, Romero R, Korzeniewski SJ, Kusanovic JP, Soto E, Lam J, et al Maternal plasma concentrations of angiogenic/antiangiogenic factors in the third trimester of pregnancy to identify the patient at risk for stillbirth at or near term and severe late preeclampsia. Am J Obstet Gynecol. 2013; 208: 287.e1–15. 23333542 10.1016/j.ajog.2013.01.016 PMC4086897

[17] Schneuer FJ, Roberts CL, Ashton AW, Guilbert C, Tasevski V, Morris JM, et al Angiopoietin 1 and 2 serum concentrations in first trimester of pregnancy as biomarkers of adverse pregnancy outcomes. Am J Obstet Gynecol. 2014; 210: 345.e1–9. 24215861 10.1016/j.ajog.2013.11.012

[18] Zeisler H, Llurba E, Chantraine F, Vatish M, Staff AC, SennstromBrennecke SP, et al Predictive Value of the sFlt-1:PlGF Ratio in Women with Suspected Preeclampsia. N Engl J Med. 2016; 374: 13–22. 10.1056/NEJMoa1414838 26735990

[19] Ferretti C, Bruni L, Dangles-Marie V, Pecking AP, Bellet D. Molecular circuits shared by placental and cancer cells, and their implications in the proliferative, invasive and migratory capacities of trophoblasts. Hum Reprod Update. 2007; 13: 121–141. 10.1093/humupd/dml048 17068222

[20] Holtan SG, Creedon DJ, Haluska P, Markovic SN. Cancer and pregnancy: parallels in growth, invasion, and immune modulation and implications for cancer therapeutic agents. Mayo Clin Proc. 2009; 84: 985–1000. 10.1016/S0025-6196(11)60669-1 19880689 PMC2770910

[21] Tamagnone L, Comoglio PM. Signalling by semaphorin receptors: Cell guidance and beyond. Trends Cell Biol. 2000; 10: 377–383. 10932095 10.1016/s0962-8924(00)01816-x

[22] Coma S, Shimizu A, Klagsbrun M. Hypoxia induces tumor and endothelial cell migration in a Semaphorin 3F- and VEGF-dependent manner via transcriptional repression of their common receptor Neuropilin 2. Cell Adhes Migr. 2011; 5: 266–275. 10.4161/cam.5.3.16294 PMC3210211 21610314

[23] Burton GJ, Jauniaux E. Placental oxidative stress: from miscarriage to preeclampsia. J Soc Gynecol Investig. 2004; 11: 342–352. 10.1016/j.jsgi.2004.03.003 15350246

[24] Redman CW, Sargent IL. Latest advances in understanding preeclampsia. Science. 2005; 308: 1592–1594. 10.1126/science.1111726 15947178

[25] Redman CW, Sargent IL. Placental stress and pre- eclampsia: a revised view. Placenta. 2009; 30: S38–42. 10.1016/j.placenta.2008.11.021 19138798

[26] Pugh CW, Ratcliffe PJ. Regulation of angiogenesis by hypoxia: role of the HIF system. Nat Med. 2003; 9: 677–684. 10.1038/nm0603-677 12778166

[27] Hirota K, Semenza GL. Regulation of angiogenesis by hypoxia-inducible factor 1. Crit Rev Oncol Hematol. 2006; 59: 15–26. 10.1016/j.critrevonc.2005.12.003 16716598

[28] Wang GL, Semenza GL. Purification and characterization of hypoxia-inducible factor 1. J Biol Chem. 1995; 270: 1230–1237. 7836384 10.1074/jbc.270.3.1230

[29] Resta L, Capobianco C, Marzullo A, Piscitelli D, Sanguedolce F, Schena FP, et al Confocal laser scanning microscope study of terminal villi vessels in normal term and pre-eclamptic placentas. Placenta. 2006; 27: 735–739. 10.1016/j.placenta.2005.07.006 16242771

[30] Maulik D, De A, Ragolia L, Evans J, Grigoryev D, Lankachandra K, et al Down-regulation of placental neuropilin-1 in fetal growth restriction. Am J Obstet Gynecol. 2016; 214: 279 e1–9. 26409917 10.1016/j.ajog.2015.09.068

