# Supplementary material for: Semaphorin 3F expression is reduced in pregnancy complicated by preeclampsia. An observational clinical study
Source: PLoS One. 2017 Mar 28;12(3):e0174400. doi: 10.1371/journal.pone.0174400 (PMC5370113; doi:10.1371/journal.pone.0174400)
Supplement: S1 Fig — Original uncropped and unadjusted blots of SEMA3F and NRP2, as shown in Fig 2A and 2B. (PPTX) [file pone.0174400.s001.pptx]

## Slide 1
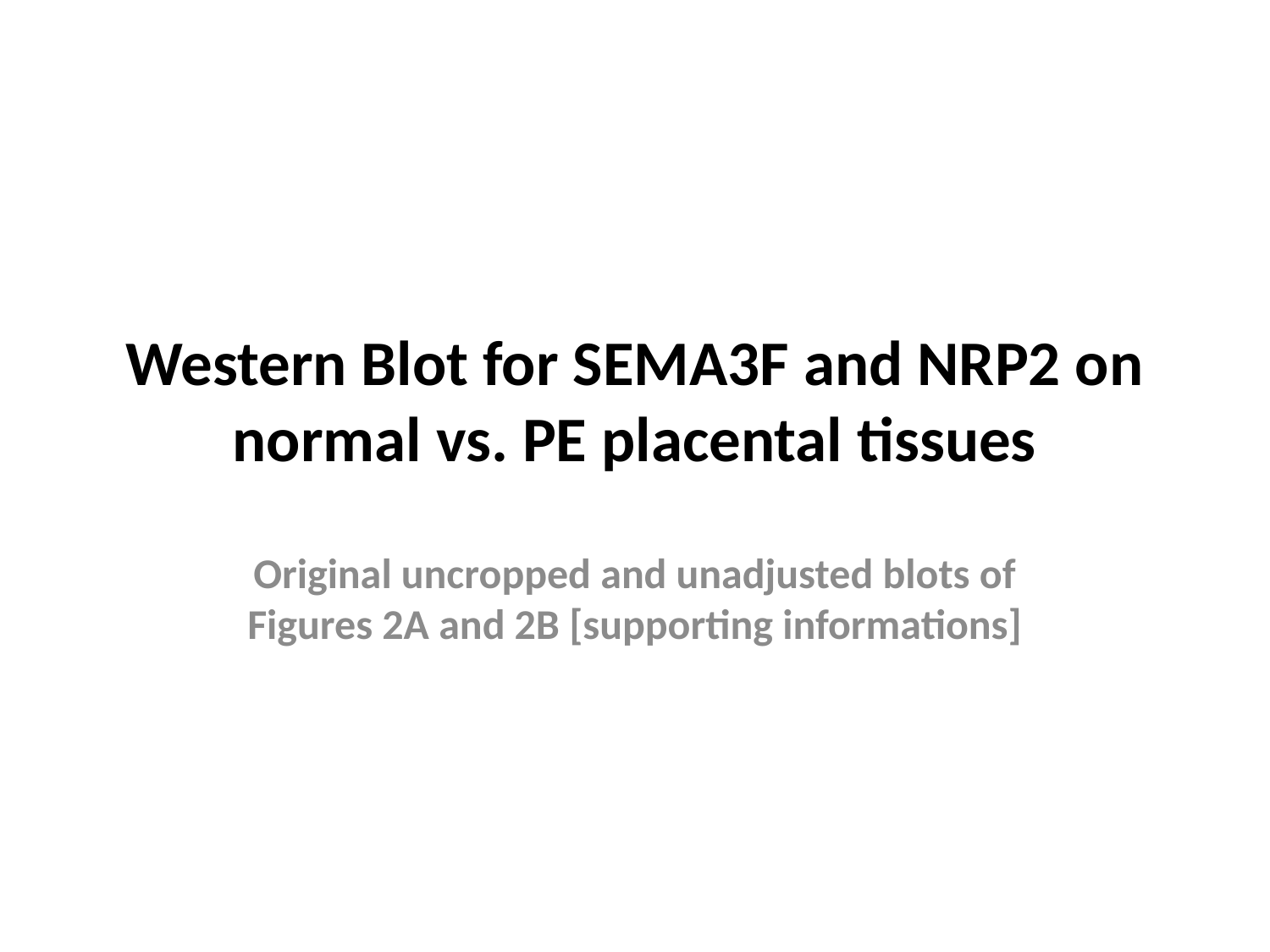

# Western Blot for SEMA3F and NRP2 on normal vs. PE placental tissues
Original uncropped and unadjusted blots of Figures 2A and 2B [supporting informations]

## Slide 2
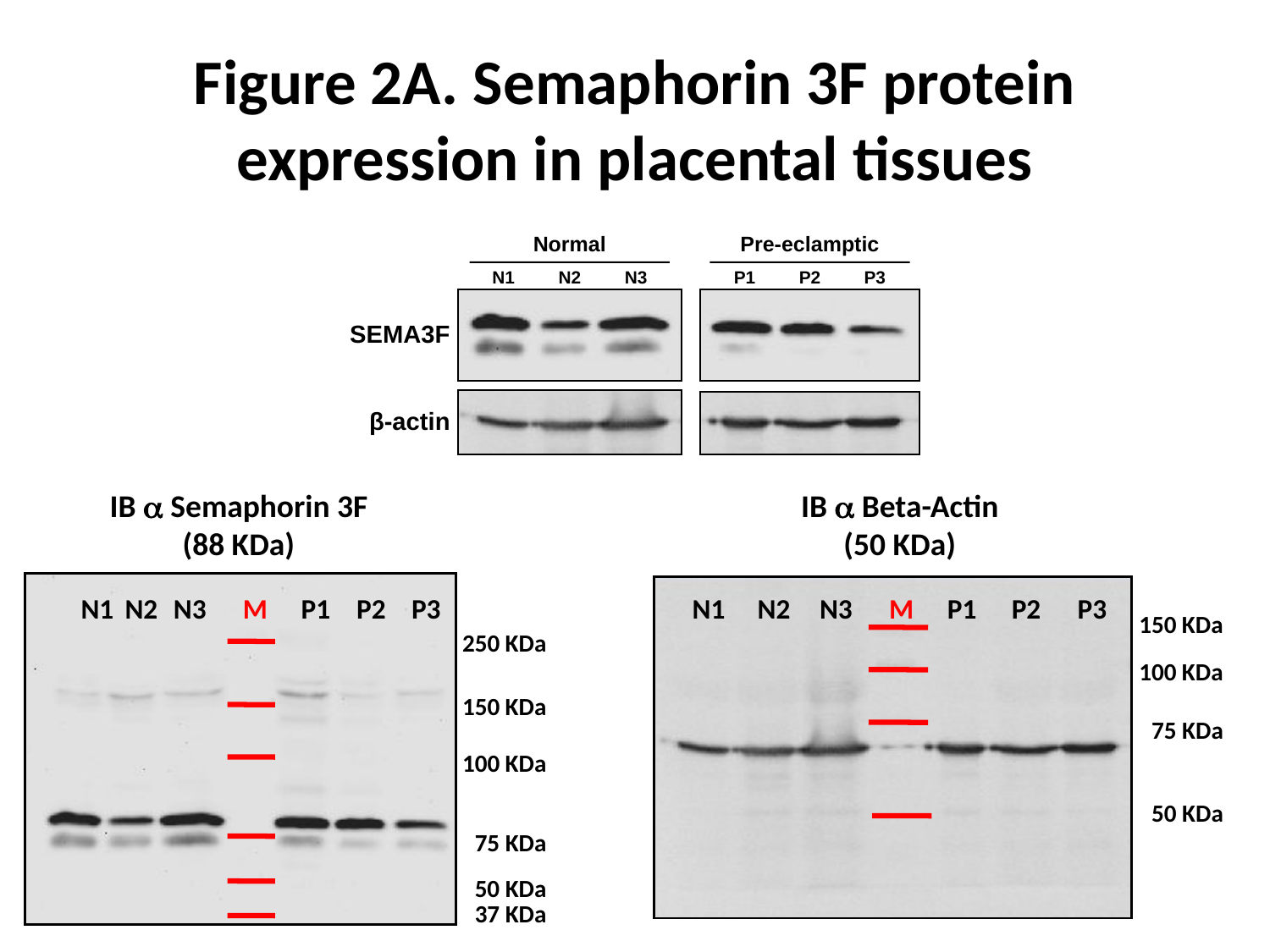

# Figure 2A. Semaphorin 3F protein expression in placental tissues
Normal
Pre-eclamptic
N1 N2 N3
P1 P2 P3
SEMA3F
β-actin
IB a Semaphorin 3F
(88 KDa)
IB a Beta-Actin
(50 KDa)
N1
N2
N3
M
P1
P2
P3
N1
N2
N3
M
P1
P2
P3
150 KDa
250 KDa
100 KDa
150 KDa
75 KDa
100 KDa
50 KDa
75 KDa
50 KDa
37 KDa

## Slide 3
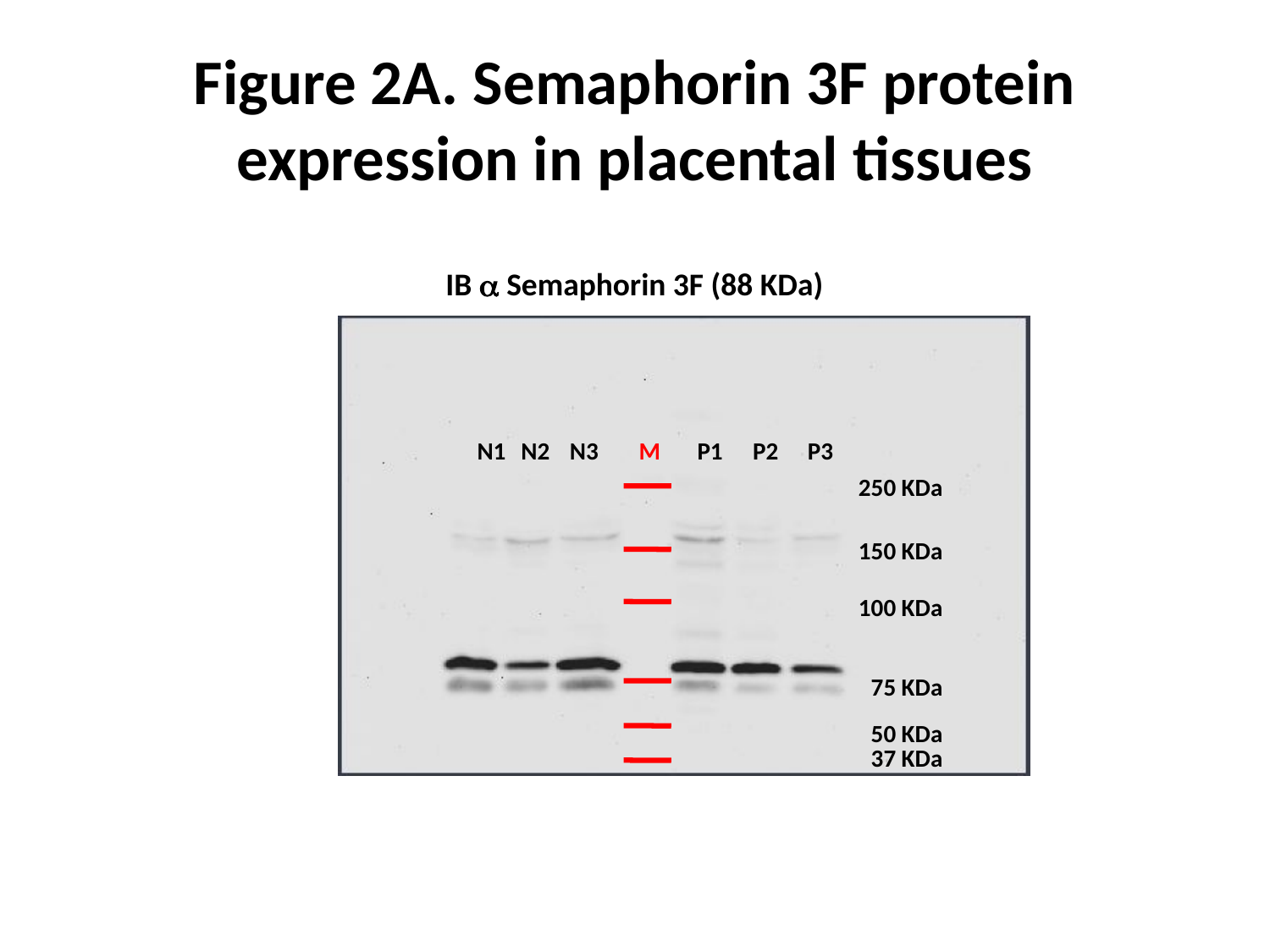

# Figure 2A. Semaphorin 3F protein expression in placental tissues
IB a Semaphorin 3F (88 KDa)
N1
N2
N3
M
P1
P2
P3
250 KDa
150 KDa
100 KDa
75 KDa
50 KDa
37 KDa

## Slide 4
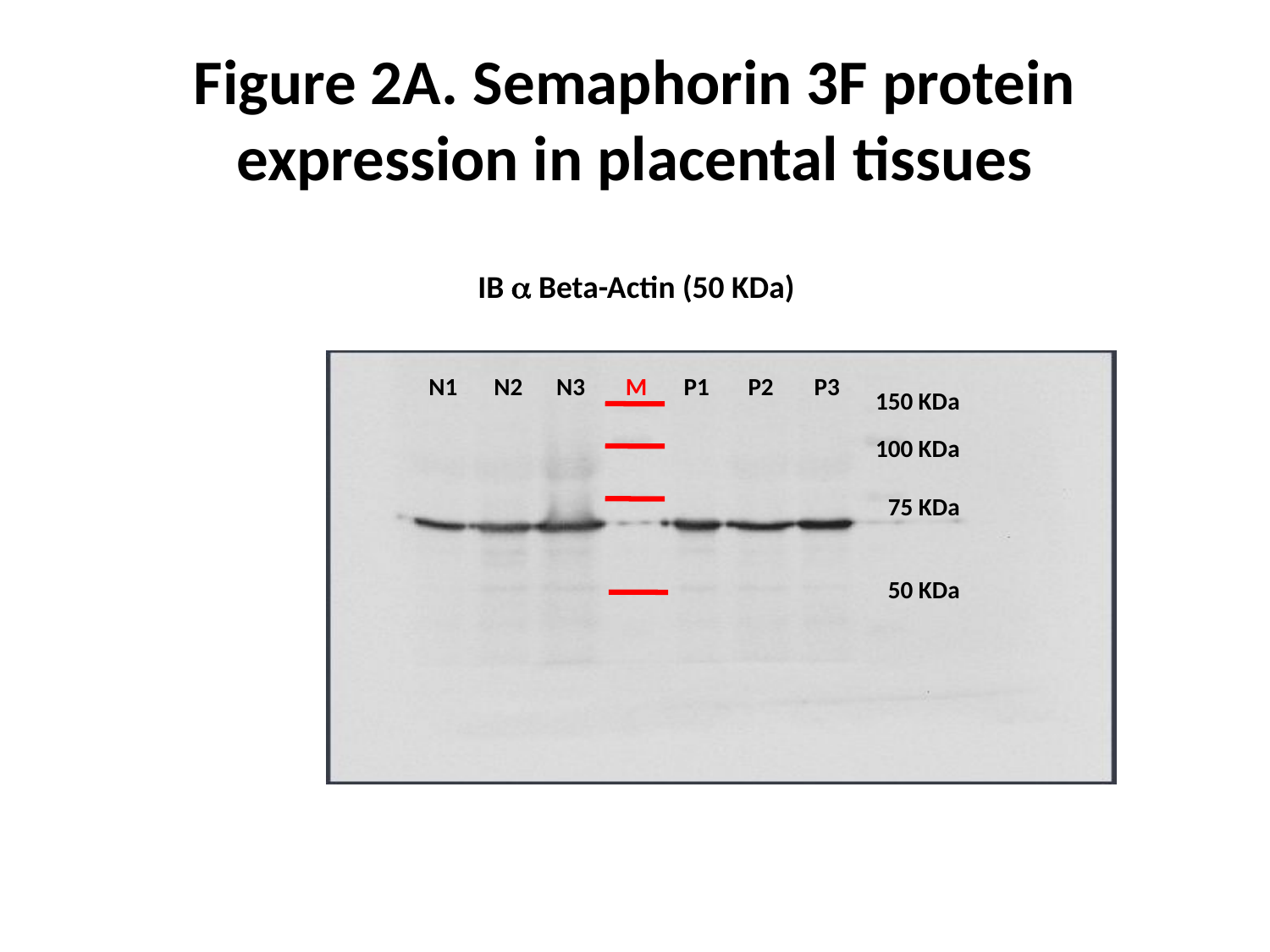

# Figure 2A. Semaphorin 3F protein expression in placental tissues
IB a Beta-Actin (50 KDa)
N1
N2
N3
M
P1
P2
P3
150 KDa
100 KDa
75 KDa
50 KDa

## Slide 5
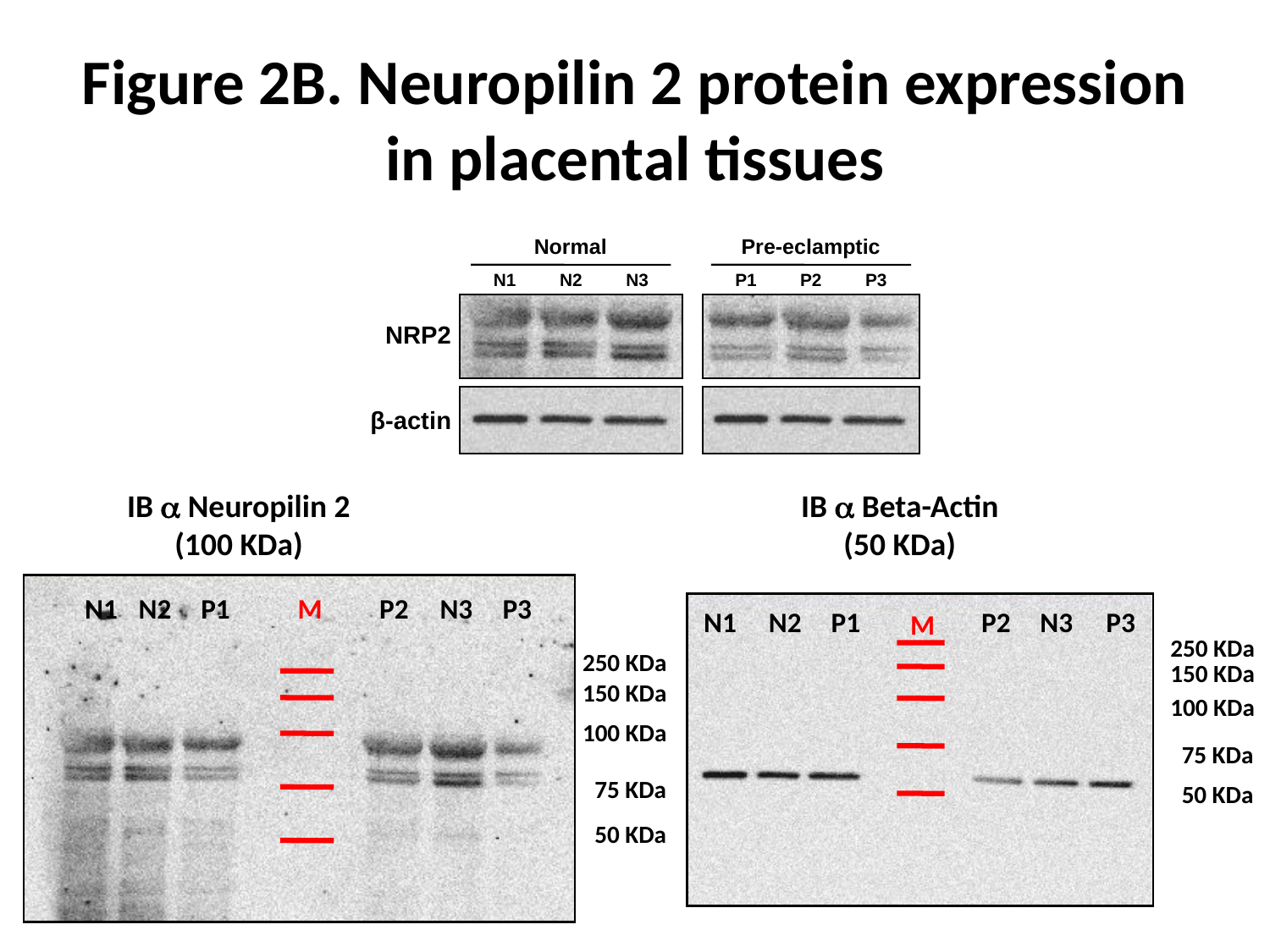

# Figure 2B. Neuropilin 2 protein expression in placental tissues
Normal
Pre-eclamptic
N1 N2 N3
P1 P2 P3
NRP2
β-actin
IB a Neuropilin 2
(100 KDa)
IB a Beta-Actin
(50 KDa)
N1
N2
P1
M
P2
N3
P3
N1
N2
P1
P2
N3
P3
M
250 KDa
250 KDa
150 KDa
150 KDa
100 KDa
100 KDa
75 KDa
75 KDa
50 KDa
50 KDa

## Slide 6
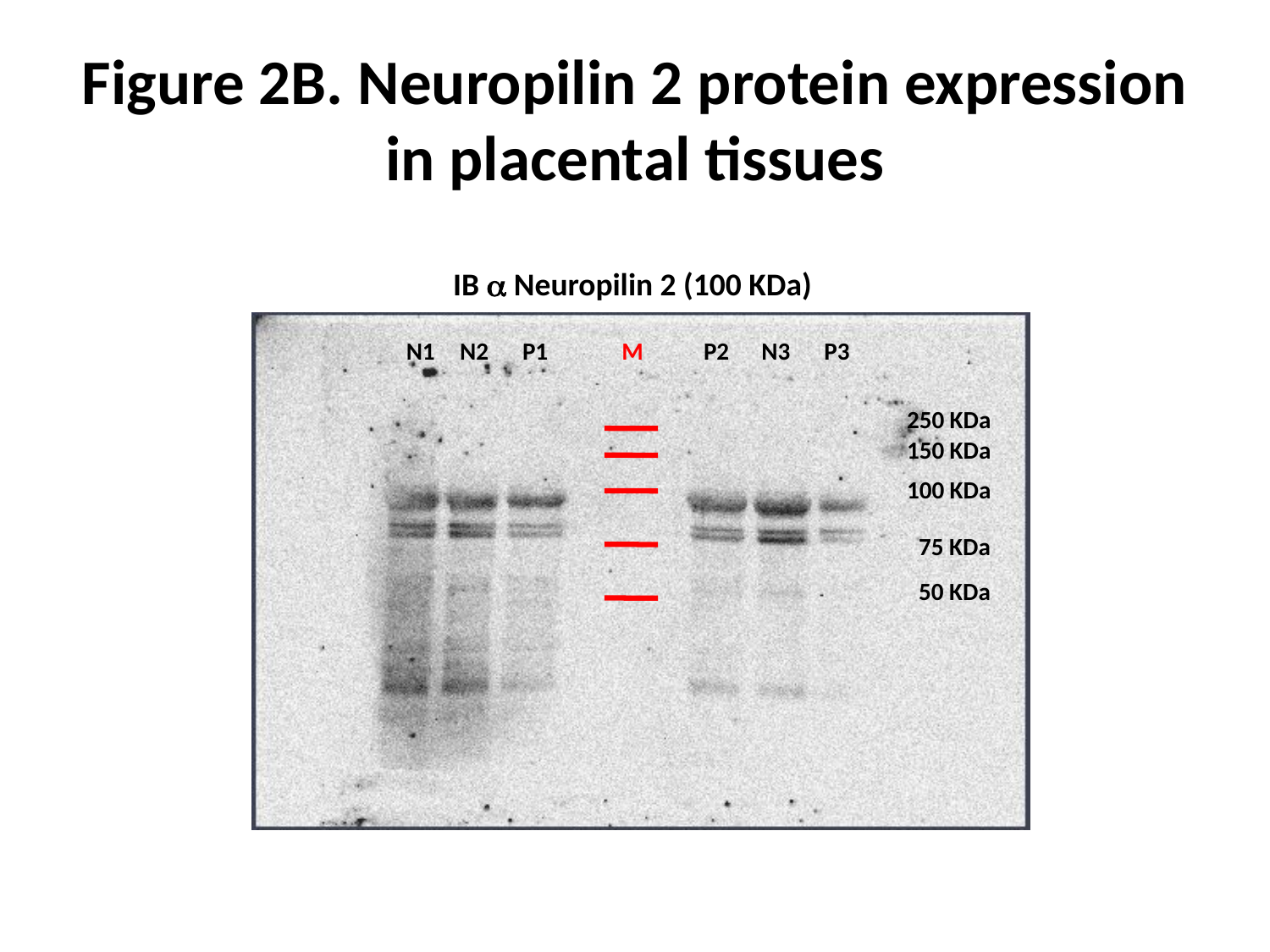

# Figure 2B. Neuropilin 2 protein expression in placental tissues
IB a Neuropilin 2 (100 KDa)
N1
N2
P1
M
P2
N3
P3
250 KDa
150 KDa
100 KDa
75 KDa
50 KDa

## Slide 7
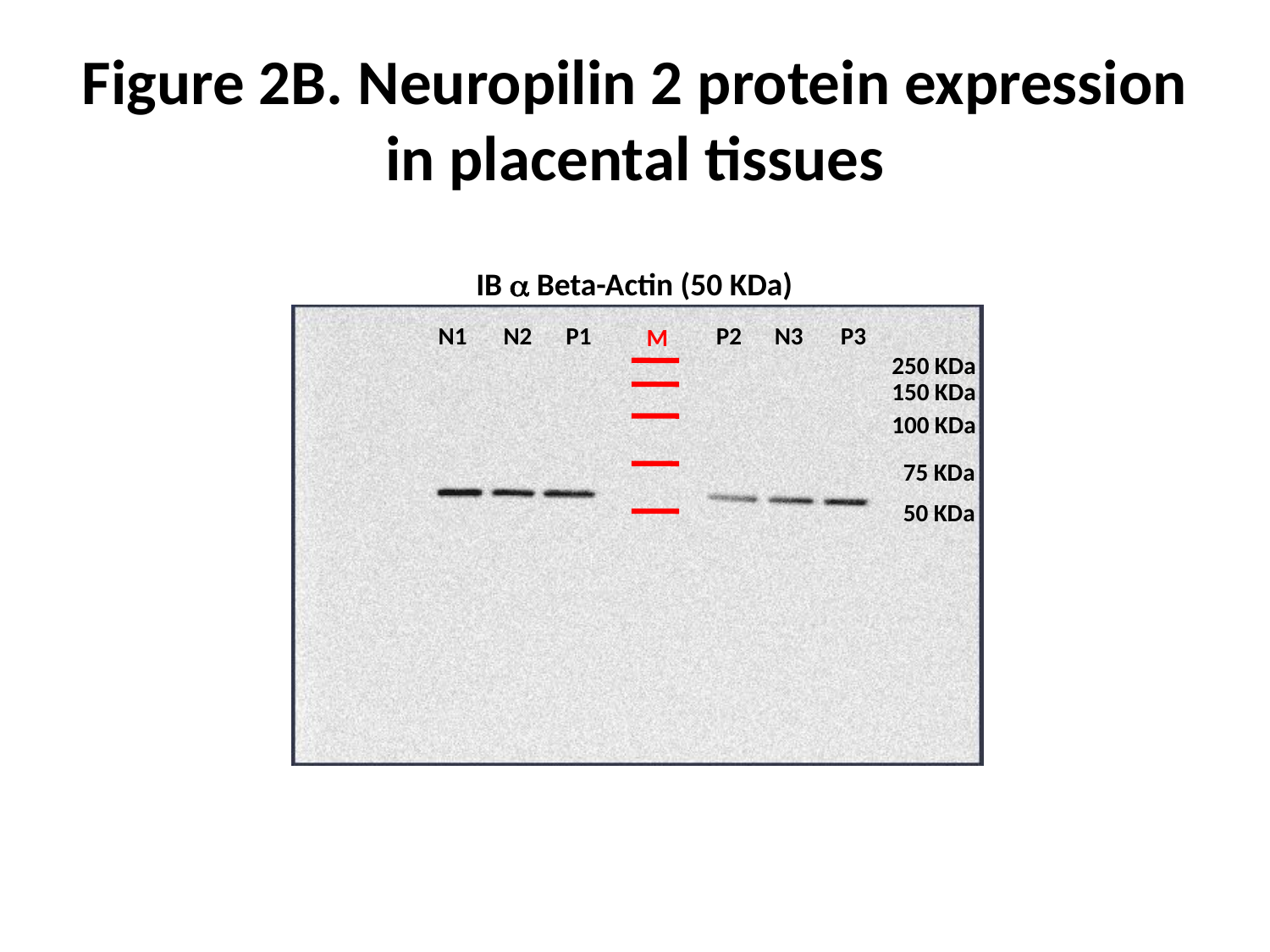

# Figure 2B. Neuropilin 2 protein expression in placental tissues
IB a Beta-Actin (50 KDa)
N1
N2
P1
P2
N3
P3
M
250 KDa
150 KDa
100 KDa
75 KDa
50 KDa
